# The Application of Supervised Machine Learning Algorithms for Image Alignment in Multi-Channel Imaging Systems

**DOI:** 10.3390/s25020544

**Published:** 2025-01-18

**Authors:** Kyrylo Romanenko, Yevgen Oberemok, Ivan Syniavskyi, Natalia Bezugla, Pawel Komada, Mykhailo Bezuglyi

**Affiliations:** 1 Department of Computer-Integrated Technologies of Device Production, Faculty of Instrumentation Engineering, National Technical University of Ukraine “Igor Sikorsky Kyiv Polytechnic Institute”, Beresteiskyi Ave., 37, 03056 Kyiv, Ukraine; kirillromanenko120700@gmail.com (K.R.); syn@mao.kiev.ua (I.S.); m.bezuglyi@kpi.ua (M.B.); 2 Department of Quantum Radiophysics and Nanoelectronics, Taras Shevchenko National University of Kyiv, 60 Volodymyrska St., 01033 Kyiv, Ukraine; oya@univ.kiev.ua; 3 Main Astronomical Observatory, National Academy of Science of Ukraine, Akademika Zabolotnoho St., 27, 03143 Kyiv, Ukraine; 4 Department of Electronics and Information Techniques, Faculty of Electrical Engineering and Computer Science, Lublin University of Technology, 38D Nadbystrzycka Street, 20-618 Lublin, Poland

**Keywords:** geometric calibration, multi-channel imaging systems, image processing, image analysis, image alignment, machine learning algorithms

## Abstract

This study presents a method for aligning the geometric parameters of images in multi-channel imaging systems based on the application of pre-processing methods, machine learning algorithms, and a calibration setup using an array of orderly markers at the nodes of an imaginary grid. According to the proposed method, one channel of the system is used as a reference. The images from the calibration setup in each channel determine the coordinates of the markers, and the displacements of the marker centers in the system’s channels relative to the coordinates of the centers in the reference channel are then determined. Correction models are obtained as multiple polynomial regression models based on these displacements. These correction models align the geometric parameters of the images in the system channels before they are used in the calculations. The models are derived once, allowing for geometric calibration of the imaging system. The developed method is applied to align the images in the channels of a module of a multispectral imaging polarimeter. As a result, the standard image alignment error in the polarimeter channels is reduced from 4.8 to 0.5 pixels.

## 1. Introduction

In modern science and technology, multi-channel imaging systems (MCISs) play a crucial role in remote sensing [1,2,3,4,5,6,7,8], medical diagnostics [9,10,11,12,13,14,15,16,17,18], computer vision, and robotics [1,19,20,21,22]. An MCIS can be implemented as a camera system [2,3,23,24], which acquires several images of the same scene using a single sensor but at different times, using different sensors at the same time, or using a single sensor where the incoming aperture is divided into multiple optical channels [4,5,25,26,27]. One primary advantage of an MCIS is the ability to simultaneously acquire more data about the objects under study [6]. As a result, it is possible to accurately determine the parameters of moving objects or transient processes and obtain more detailed images of objects, enhancing the accuracy and efficiency of the acquired data [6].

Many tasks require the joint analysis of two or more images of the same scene acquired in the channels of an MCIS (see, e.g., [7,8,10,11,12,13,14,15,16,17]). A significant challenge in data processing within an MCIS is resolving the geometric distortions and, consequently, the geometric inequalities between the images obtained in the channels [28,29]. Differences in images can arise due to the detector’s geometric size, differences in the optical paths of light within the channels, discrepancies in optical element parameters across channels, and other factors [29,30]. This can lead to substantial errors when analyzing the geometric boundaries and the physical characteristics of the studied phenomena and objects [27,31,32]. Therefore, an MCIS requires high-precision image alignment across channels.

In general, image alignment establishes a correspondence between the coordinate system of one reference image (real or modeled) and another. Image alignment in MCIS channels ensures their consistency, without which reliable and accurate analysis of the parameters and characteristics of scenes studied using the MCIS is impossible.

Based on the type of geometric distortion in the images acquired in the system channels, different effective approaches to image alignment have been proposed and used. Here, we select three representative cases. The first occurs when the images in the system channels are affected by linear distortion (shifts, rotations, scaling, skews). The second case considers the radial distortion of images in the channels. The last is the common occurrence of an arbitrary non-linear distortion of images in the channels of an MCIS.

In the linear distortion case, area- and feature-based techniques are the most effective for image alignment. In these techniques, the alignment process usually consists of a few steps. First is the selection of features in the images (areas, lines, edges, line intersections, or well-selectable regions). In the second step, each feature in one image is compared with potentially corresponding features in the other image. Pairs of points representing the centers (centers of gravity) of the selected features with similar attributes are defined as matches. The set of these points is called the control points. In the final step, the set of obtained control points is used to estimate the parameters of the transformation that best models the deformation between images [7,8,33,34,35]. The similarity of the selected image features has been estimated using approaches based on the normalized cross-correlation [34,36], Fourier transformation difference [33,34,37], binary correlation [8,38], chain-code correlation, distance of invariant moments, or structural matching [8]. As an example, in [8], for scenes of around 512 × 512 pixels, depending on the number of control points in the experiments, the described techniques demonstrated an image registration (alignment) accuracy of less than 0.3 pixels at each control point, with a root mean square (RMS) error of less than 0.2 pixels obtained for the 10 control points.

Some key advantages of these techniques are that they do not require special stands or patterns for geometric calibration, and the search for control points can be automated. Additionally, the use of supervised machine learning algorithms allows for optimization of the time spent searching for control points and image alignment [7]. Meanwhile, a disadvantage is that the techniques are mainly well suited for the alignment of images with linear distortions. In the case of radial distortion, it can be effectively described within the scope of a simple polynomial model of order three or five [11,39,40]. The polynomial coefficients are usually calibrated using different test boards or templates. In [41], for example, a chessboard template was used as a test board. A two-step calibration method was then proposed based on global polynomial projection model fitting (first step) and local line fitting optimization (second step). For a 1280 × 1024 pixel scene, the largest reprojection error (interpreted as the minimum image alignment accuracy) was limited to 0.90 pixels, with an average error of 0.15 pixels and an RMS error of 0.007 pixels. The reprojection error was defined in [41] as the difference between the actual coordinates of the point in the image and the corresponding coordinates of the projected point through a projection model in the reference image. In [40], for the geometric calibration of a wide-angle camera objective, an optical reference generator was proposed, which consisted of 28 collimators whose viewing directions are well known. The accuracy of the light spot coordinates produced by the collimators on the image sensor and the incident directions provided the parameters for the geometric model using a least squares algorithm. The calibration accuracy of the described approach is very high: the absolute location of image points on the 242 × 274 pixel detector was within 0.05 pixels. However, to calibrate different sensors, a new reference generator must be designed. In [42], a more universal optical reference generator was proposed based on the use of a single collimator mounted on a two-dimensional turntable. In [39], instead of precisely adjusting the position of the sensor on the turntable, a rotation matrix was introduced. This effectively eliminated the errors caused by the mechanical axis of the turntable and the optical axis of the sensor not being adjusted to the same direction through the rotation transformation of the coordinate system. As a result, the calibration residual of the proposed method was less than 0.1 pixels with an RMS error of 0.07.

The advantage of the above techniques is that they allow for the alignment of images with radial distortion with a very high potentially achievable precision. This is due to the simple, well-studied pattern characterizing distortion of this type. Additionally, in this case, simple models and simple test patterns can be used for calibration. On the other hand, the abovementioned techniques are suitable only for radial distortion cases. However, they can be used as stages in multi-stage alignment techniques for images with non-radial, non-linear distortions (see, e.g., [43]).

Apart from the differences in the linear dimensions of images in MCIS channels—which are primarily eliminated through precise mutual alignment of the channels [26]—various types of non-linear non-radial distortions are possible, such as those in wide-angle systems [43]. In [43], the non-linear distortion of an ultra-wide-angle imaging system was presumed to be the result of a combination of radial and perspective distortions. The proposed technique for distorted image alignment (distortion compensation) at the first stage compensated for the radial aspect of the distortion, according to the radial distortion model. Next, perspective distortion correction was carried out. Achievable image correction errors were in the range of 1–3 pixels (depending on the angular position of the camera relative to the planar grid template). The errors were estimated for images obtained with an ultra-wide-angle lens (Pentax K-x camera with Pentax DA Fish-Eye 10–17 mm F3.5–4.5 ED lens, Pentax Corporation, Tokyo, Japan) at an image resolution of 3072 × 2048 pixels.

An advantage of the above approach is that it considers a more complicated non-linear model of distortion that includes both radial and perspective distortions. However, in general, distortion cannot be reduced to a combination of only these two types; thus, improving existing approaches and developing new, more general approaches for image alignment in multi-channel systems with non-linear distortion are necessary.

The presence of non-linear distortions in images significantly complicates their geometric alignment. One way to ensure consistent positioning and representation of objects in MCIS channels is to use machine learning algorithms for image alignment [44,45].

Machine learning algorithms are computational algorithms or mathematical models that enable computers to learn from data and make predictions or decisions without explicit programming [46]. These algorithms learn by analyzing datasets to identify and explore patterns, relationships, and statistical dependencies. Machine learning algorithms can be classified into various types based on their learning approaches and objectives. Supervised learning algorithms learn from labeled training data, where input data are paired with corresponding output or target labels [46]. The primary goal of machine learning algorithms is to generalize from training data and apply the acquired knowledge to new or unseen data [46]. They employ a variety of approaches, including linear and non-linear models [47], allowing for the effective identification of patterns characterized by non-linear dependencies. The main components include training data to build models, evaluation methods to verify model performance, and optimization algorithms for tuning model parameters [48]. Machine learning algorithms facilitate the automation of decision-making and prediction processes [49], ensuring high accuracy and speed in data processing, which are essential for real-world applications.

This study proposes the use of a multiple polynomial regression model as a supervised machine learning approach. This model allows for the determination of the dependency between non-linear geometric distortions in images from MCIS channels and the alignment of these images. This is crucial for determining the correct correlations between channels and maintaining consistency in the subsequent analysis of the studied object.

In fact, image alignment allows for the establishment of a correspondence between the pixel coordinates of one image and those of another [50]. Image alignment in MCIS channels ensures their consistency, without which reliable and accurate analysis of the parameters and characteristics of the scenes studied is impossible.

The image alignment method for the MCIS proposed in this study is based on the application of image pre-processing methods and machine learning algorithms, using a calibration stand with a set of ordered markers at the nodes of an imaginary grid. Furthermore, the software implementation of the data processing algorithms using the Python programming language allows for the automation of the alignment process.

## 2. Proposed Image Alignment Method

In the proposed method, one of the MCIS channels is used as a reference. The coordinates of the marker centers are determined on the calibration stand images in the system’s channels. Subsequently, the displacements of the marker centers in all channels of the system relative to the coordinates of the markers for the reference channel are determined. Based on the obtained displacements, corrective models are derived to align the geometric parameters of the images in the reference and other channels. Thus, the image alignment method includes the following stages:

- Pre-processing of the input calibration images.
- Determination of the coordinates of the marker centers in calibration stand images.
- Obtaining corrective models.
- Image alignment in the channels.
- Assessment of the alignment accuracy of images from different optical channels.

This universal approach allows, for example, a synthesized calibration image with precisely defined marker coordinates at the calibration grid nodes to be used as a reference.

### 2.1. Pre-Processing of the Input Calibration Images

The proposed method involves pre-processing these images to enhance the accuracy of the marker center coordinates on the calibration stand images in the MCIS channels.

Color images must be converted to grayscale using a standard formula that incorporates weighted coefficients for each color in the RGB scheme as follows [51]:

Y ← 0.299 × R + 0.587 × G + 0.114 × B,(1)

where R, G, and B denote the red, green, and blue color channels, respectively.

Further, Gaussian filtering is used to remove noise from the image. Gaussian filtering is an important step in image processing as it reduces the impact of noise and artifacts, which can distort the results of subsequent analysis. Noise is often present in images due to various factors, such as camera hardware imperfections or shooting conditions [52]. Gaussian filtering allows for smoothing of the image and improving the accuracy of edge detection [53]. The application of the Gaussian filter is optimal as it gently blurs the image while preserving important features, particularly object edges [53]. The mathematical expression for the filter is as follows [54]:

(2) $$G\left(x,y\right)=\frac{1}{2\pi \sigma^{2}}e^{\left(-\frac{x^{2}+y^{2}}{2\sigma^{2}}\right)},$$

where *σ* is the variance of the Gaussian filter.

To isolate the bodies of markers, the input image undergoes binarization—a process that converts it into a binary image, where each pixel’s intensity assumes one of two possible values: black or white. This transformation, also known as thresholding, is a fundamental step in many image processing procedures involving object edge detection. By transforming grayscale images into binary form, binarization simplifies further analysis by distinctly separating the details from the background. The image thresholding is performed according to the condition [55]:

(3) $$g\left(x,y\right)=\left\{\begin{array}{l} 1,\;\;\mathrm{if}\;f\left(x,y\right)\geq \;I_{n}\\ 0,\;\;\mathrm{otherwise} \end{array}\right.,$$

where $g\left(x,\;y\right)$ is the output intensity pixel value, $f\left(x,\;y\right)$ is the input intensity pixel value, and $I_{n}$ is the threshold intensity value.

By pixel intensity, we mean its grayscale shade. Thresholding allows for retaining only those image pixels that belong to the marker bodies, which are necessary for subsequent image analysis stages. The Canny edge detector (CED) is applied to obtain the edges of each marker in the image frame. The Canny edge detector is an operator that uses a multi-stage algorithm to detect edges in a wide range of images [56].

The smoothed image is filtered using a Sobel kernel in both the horizontal and vertical directions in order to obtain the first-order partial derivative in the horizontal ($G_{x}$) and vertical ($G_{y}$) directions. From these two derivatives, the edge gradient and direction for each pixel are obtained as follows [57]:

(4) $$G=\sqrt{G_{x}^{2}+G_{y}^{2}},$$

(5) $$\theta =arctg\left(\frac{G_{y}}{G_{x}}\right).$$

The gradient direction *θ* is always perpendicular to the edges. It is rounded to one of four angles representing vertical, horizontal, and two diagonal directions [58]. As a result, the contours of all markers on the calibration stand image are determined.

### 2.2. Definition of the Coordinates of Marker Centers on Images of a Calibration Stand

Applying Gaussian filtering to an image can lead to the displacement of object edges [54]. Therefore, a rectangular area is outlined around each obtained label contour in order to fully encompass the label for determination of the coordinates of its center on the calibration stand image. This step is important for the precise identification and localization of the center of each label in the image. The dimensions of this area are chosen to fully cover the marker, ensuring that its entire structure is captured without including excessive surrounding regions that could affect the calculation accuracy. By outlining the defined rectangular area, the algorithm can more effectively focus on the localized area to determine the label’s center. The size of the area encompassing the label is expressed as follows:

(6) $$x_{min}-5<x\leq x_{max}+5,$$

(7) $$y_{min}-5<y\leq y_{max}+5,$$

where $x_{min},\;y_{min}$ are the minimum values of the marker’s contour coordinates in the *x* and *y* directions, respectively; and $x_{max},\;y_{max}$ are the maximum values of the marker’s contour coordinates in the *x* and *y* directions, respectively.

The coordinates of the marker center are calculated as the coordinates of the mass center, $x_{c}\;and\;y_{c}$, on the corresponding rectangular region, weighted by the pixel intensities within that region of the image. The intensity of each pixel acts as a weighting factor, meaning that pixels with higher intensity have a greater influence on the center’s position, ensuring accurate calculation even under non-uniform lighting conditions. The center is determined by summing the products of pixel coordinates and their intensities, normalized by the total intensity. This approach helps to maintain accuracy in marker localization. The calculation formula is as follows [39]:

(8) $$x_{c}=\frac{\sum_{x,y}x\cdot I(x,y)}{\sum_{x,y}I(x,y)},$$

(9) $$y_{c}=\frac{\sum_{x,y}y\cdot I(x,y)}{\sum_{x,y}I(x,y)},$$

where $I\left(x,y\right)$ represents the intensity value (gray shade) of the pixel at coordinates (x,y).

The calculated centers of markers on the reference channel image serve as benchmarks against which subsequent adjustments in marker coordinates are calculated for other channels in the MCIS.

### 2.3. Obtaining Correction Models

The calculated coordinate displacements of markers in the MCIS channels relative to the reference channel images can be approximated using respective correction models, which involve multiple polynomial regressions trained on the obtained displacements of marker centers from the reference channel. These models determine the displacements of any pixel’s coordinates in the current MCIS channel relative to the reference channel. Each MCIS channel is generally described by its own set of correction models, separately obtained for *x* and *y* coordinates.

In turn, the correction model utilizes multiple polynomial regression to model the relationship between the dependent and independent variables. The polynomial formula can be described as follows:

(10) $${u=a}_{0}+\sum_{n=1}^{N}{\left(a_{n}x+b_{n}y\right)}^{n},$$

where x, y are independent variables (pixel coordinates);$\;a_{n},\;b_{n}$ are constant coefficients for variables *x* and *y*, respectively; *u* is the dependent variable (correction offset);$\;a_{0}$ is the zero-degree coefficient; *n* is a positive integer; and *N* is the order of the polynomial.

After obtaining the correction model, its accuracy is validated using test data separated from the data used to build it. The root mean square error (RMSE) is calculated to assess the model’s accuracy. Additionally, coefficients of determination (R^2^) are computed to evaluate how well the model predicts results for each obtained correction model. The polynomial order can be chosen based on the complexity of distortions and the required precision to align geometric parameters across the MCIS channels. For instance, cubic polynomials [39,40,42] are commonly used to compensate for pillow-like distortion in wide-angle imaging systems.

The obtained models calculate correction displacements separately for the *x* and *y* axes and for each pixel in the MCIS image relative to the reference channel. For example, consider an MCIS where Img_ref is the image obtained in the reference channel and Img_cur is the image in the adjacent channel. During geometric calibration for the adjacent channel, correction models are obtained: *u*(*x*,*y*) for the *x*-axis and *v*(*x*,*y*) for the y-axis. Therefore, a portion of the input scene that is projected by the MCIS optical system—for instance, onto a pixel at coordinates (100, 100) in Img_ref of the reference channel—will be projected by the optical system of the adjacent channel onto a pixel at coordinates (100 + *u*(100,100), 100 + *v*(100,100)) in Img_cur.

### 2.4. Image Alignment in the Channels

One difficulty in the alignment of images is that the polynomials underlying the model are continuous functions, and corrections predicted by them often result in non-integer values. For example, to correct the working frame of an image using a correction model, pixels at coordinates (128, 300), (129, 300), and (130, 300) in the corrected image may correspond to pixels from the working image at coordinates (126.1, 300.3), (126.3, 300.4), and (126.5, 300.5). Simply rounding or discarding the fractional part of these coordinates will duplicate the pixel from the working image in several locations of the corrected image. Such duplication reduces the accuracy when analyzing the parameters of the observed scene (e.g., intensity distribution, spectrum, polarization) in the input of a multi-channel system.

Using the intensity values obtained through bilinear interpolation of neighboring pixels on the working image is proposed to mitigate the issue of pixels with non-integer displacements in corrected image frames. That is, for coordinates in the working image such as (126.1, 300.3), (126.3, 300.4), and (126.5, 300.5), the respective weighted intensities are calculated from a bilinear interpolation function constructed using intensities from pixels at coordinates (126, 300), (126, 301), (127, 300), and (127, 301).

The bilinear interpolation process considers the intensity values of neighboring pixels, which play a crucial role in returning a weighted value. Initially, we linearly interpolate in the *x*-axis direction [59]:

(11) $$R_{1}=\frac{x_{2}-x}{x_{2}-x_{1}}\times I\left(x_{1},y_{1}\right)+\frac{x-x_{1}}{x_{2}-x_{1}}\times I\left(x_{2},y_{1}\right),$$

(12) $$R_{2}=\frac{x_{2}-x}{x_{2}-x_{1}}\times I\left(x_{1},y_{2}\right)+\frac{x-x_{1}}{x_{2}-x_{1}}\times I\left(x_{2},y_{2}\right),$$

where $I\left(x_{n},y_{m}\right)$ is the intensity value of the pixel.

Next, we perform interpolation in the *y*-axis direction (between points $R_{1}$ and $R_{2}$) using the following formula [59]:

(13) $$P=\frac{y_{2}-y}{y_{2}-y_{1}}\times R_{1}+\frac{y-y_{1}}{y_{2}-y_{1}}\times R_{2}.$$

### 2.5. Estimation of Image Alignment Accuracy in Optical Channels

The accuracy of pixel alignment in the MCIS channels is evaluated by predicting the displacements of each pixel’s coordinates from their proper position. For this purpose, a set of test data representing actual displacements for some marker centers is isolated during the training of correction models. This test data set is used to assess the accuracy of the obtained correction models. As the proper displacement value for such a test data set is known, we can compare it with the predicted value obtained through the model and calculate this value as the root mean square error using the following formula [60]:

(14) $$RMSE=\sqrt{\frac{1}{n}\sum_{i=1}^{n}{\left(y_{i}-\hat{y_{i}}\right)}^{2}},$$

where $\hat{y_{i}}$ is the predicted value, $y_{i}$ is the actual value, and *n* is the total sample size.

Next, we obtain the coefficient of determination $R^{2}$, which assesses how well the model predicts the results. The coefficient of determination ranges from 0 to 1, where 0 indicates that the model does not explain the variability of the data, and 1 indicates that the model perfectly predicts all values [61]. The coefficient of determination is calculated on the test data using the following formula [61]:

(15) $$R^{2}=\frac{SST-SSE}{SSE},$$

where *SSE* denotes the sum of squared errors and *SST* is the sum of squares.

Moreover, the accuracy of pixel location correction in the channels after alignment is assessed through a comprehensive analysis of the aligned images. The centers of the markers are precisely determined in the aligned channel images, and the standard deviation of these centers from the reference values taken from the reference image frame is calculated with great care. These values are computed using the formula [62]:

(16) $$\sigma =\sqrt{\frac{\sum_{i=1}^{n}{\left(x_{i}-\bar{x}\right)}^{2}}{n-1}},$$

where $x_{i}$ is the deviation of center value for marker *i*, $\bar{x}$ is the mean value of all deviations, and n is the number of marker centers.

The functionality represented by expressions (1)–(5), (10), and (14)–(16) was implemented in the OpenCV library [63], designed for image processing, and in Scikit-learn [64], designed for machine learning algorithms, both of which are available for Python.

## 3. MCIS Image Alignment Algorithm

**Figure 1:**
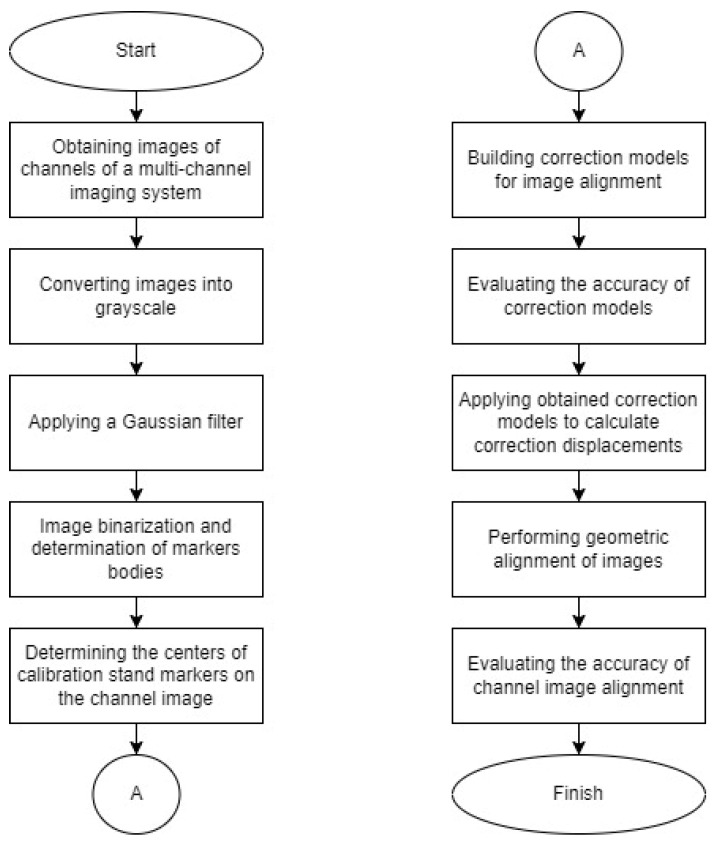
Algorithm for image alignment using the proposed method.

The process of image alignment using the described method begins with obtaining images of a calibration stand in the channels of an MCIS (Figure 1). The calibration stand typically consists of a panel with orderly arranged visual elements, such as markers (e.g., grids, measures, chessboards, shapes of specified forms, and point light sources) [32,39,40,42,65,66], which are easily distinguishable and can be localized in the images of this panel in the channels of the imaging system.

The original image of the calibration stand may contain color information that is not essential for detecting markers in the image. Therefore, the image is first converted to grayscale, simplifying subsequent analysis by transforming the three color channels (RGB) into a single intensity channel, thereby reducing computational complexity. Images often contain noise and artifacts that can lead to false marker detections during binarization. To address this, the grayscale image is then passed through a Gaussian filter. The Gaussian filter is chosen as it effectively reduces high-frequency noise, smooths the image, and enhances the accuracy of subsequent edge detection. This step is important for improving the quality of the binarization process.

To separate marker bodies from the background, the filtered grayscale image is binarized. Binarization converts the image into a binary format where pixels are either black or white, representing the foreground (markers) and the background, respectively. The binary image is analyzed to determine the edges of each marker. These edges are then enclosed within rectangular bounding zones to ensure complete coverage. This step is necessary as the Gaussian filtering process may alter the edges of each marker, and binding them with rectangles ensures that the marker will be fully analyzed in subsequent steps. Next, the coordinates of the marker centers within the extracted rectangles are calculated as the centroids of pixel intensity within the enclosed area. Accurate localization of the marker centers is important for subsequent calculations of displacements and correction of deviations.

Based on the calculated centers of each marker in the image, deviations from the reference positions on the baseline frame are determined. Using these deviations, individual correction models are constructed for each frame. Subsequently, the accuracy of each model is evaluated. For this purpose, the data set is split into training and validation sets, ensuring that the models are tested on data not used during training. To assess the model’s performance, the root mean square error (RMSE) between the predicted and actual deviations is calculated.

The validated models are applied to the entire set of pixel coordinates in the channels to compute the corrected positions. This involves adjusting the original coordinates of each pixel based on the predicted offsets derived from the correction models. Applying corrections to all pixels ensures that the entire image is properly aligned, not just the positions of the markers.

The accuracy of image alignment within the MCIS channels is assessed by calculating the standard deviation of the marker center coordinates on the calibration stand nodes from their reference positions in the selected frame of the reference image.

## 4. Results

After the described algorithm was implemented in Python, it was applied for image alignment in a multispectral imaging polarimeter (MSIP) [67]. The main objective of the MSIP is to measure the distribution of the intensity and polarization state of scattered light across the observed scene in a wide 60° × 60° field in a common wavelength range of 370–1700 nm. To achieve this objective, the MSIP combines five modules that are equivalent in design. Each module is a narrowband polarimeter/photometer operating in a specific wavelength range. In conjunction, all modules provide the multispectral capability of the MSIP.

**Figure 2:**
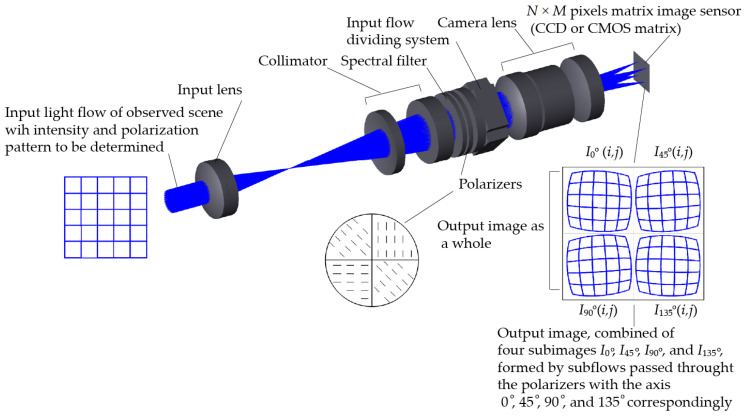
Simplified common optical scheme of MSIP modules.

A simplified common optical scheme of MSIP modules is shown in Figure 2. One module consists of the input lens system, collimator, combined polarizer, spectral filter, dividing system, camera lens, and matrix image sensor. The input lens system directs an image of the observed scene through the combined polarizer and narrowband spectral filter to a system that divides the input flow into four parallel, spatially separated equivalent subflows (channels). Each subflow carries a copy of the input image. The combined polarizer consists of four segments of arranged polarizing films with polarizing axis orientations of 0°, 45°, 90°, and 135°. Each subflow passes through the corresponding segment of the combined linear polarizer before falling on the sensor with the specified orientation of the polarizing axis. Through falling on specified subareas of the single matrix image sensor (CCD or CMOS matrix), the subflows form a single output image, as shown in Figure 2.

Thus, the final image on the MSIP module image sensor is a combination of sub-images $I_{0{}^{\circ}}$, $I_{90{}^{\circ}}$, $I_{45{}^{\circ}}$, and $I_{135{}^{\circ}}$ (see Figure 2), whose pixel brightness values $I_{0{}^{\circ}}\left(i,j\right)$, $I_{45{}^{\circ}}\left(i,j\right)$, $I_{90{}^{\circ}}\left(i,j\right)$, and $I_{135{}^{\circ}}\left(i,j\right)$ are used to obtain the distribution of light intensity and polarization across the observed scene. These are described using the so-called Stokes parameters I, Q, and U [68], which are related to the above pixels’ brightness in the following manner:

(17) $$\;I\left(i,j\right)=I_{0{}^{\circ}}\left(i,j\right)+I_{90{}^{\circ}}\left(i,j\right)=I_{45{}^{\circ}}\left(i,j\right)+I_{135{}^{\circ}}\left(i,j\right),\;Q\left(i,j\right)=I_{0{}^{\circ}}\left(i,j\right)-I_{90{}^{\circ}}\left(i,j\right),\;U\left(i,j\right)=I_{45{}^{\circ}}\left(i,j\right)-I_{135{}^{\circ}}\left(i,j\right).$$

Once determined, the Stokes parameters (17) can be used to estimate the distribution of intensity, degree of linear polarization, and angle of linear polarization of scattered light [66] across the observed scene, when necessary.

In (17), i,j are the indices of sub-image pixels. It is clear from Figure 2 that, for the image sensor with resolution *N* × *M*, the sub-images $I_{0{}^{\circ}}$, $I_{90{}^{\circ}}$, $I_{45{}^{\circ}}$, and $I_{135{}^{\circ}}$ should have at least a twice lower resolution (*N*/2 × *M*/2). The actual sub-image resolutions are further reduced in order to avoid overlapping at the image sensor.

Measurements using the MSIP module are carried out as follows:

- A combined image of the observed scene is directly captured by the image sensor of module;
- The captured image is divided into sub-images $I_{0{}^{\circ}}$, $I_{90{}^{\circ}}$, $I_{45{}^{\circ}}$, and $I_{135{}^{\circ}}$;
- The brightness values of the sub-image pixels are substituted into (17), and the Stokes parameters are calculated at each pixel of the input scene with the final resolution determined by the sub-image resolution;
- The intensity, degree, and azimuth distributions of the polarizations across the observed scene are estimated, if necessary.

Note that the direct use of Equation (17) is correct only if the geometric parameters of the sub-images on the MSIP module sensor are absolutely identical. At the same time, imperfections in the lens and dividing systems of module can create inequalities between the geometries of the sub-images (see the output image in Figure 2, where the difference in image geometries in the channels is slightly exaggerated for clarity). To ensure the necessary accuracy in determining the distributions of the polarization parameters across the input scene when applying Equation (17), the sub-images in the MSIP module require geometric alignment.

Essentially, each module of the MSIP is a four-channel imaging system, where the channels are formed by spatially separated light subflows. Therefore, the MSIP modules are ideal for testing the algorithm described in this work.

**Figure 3:**
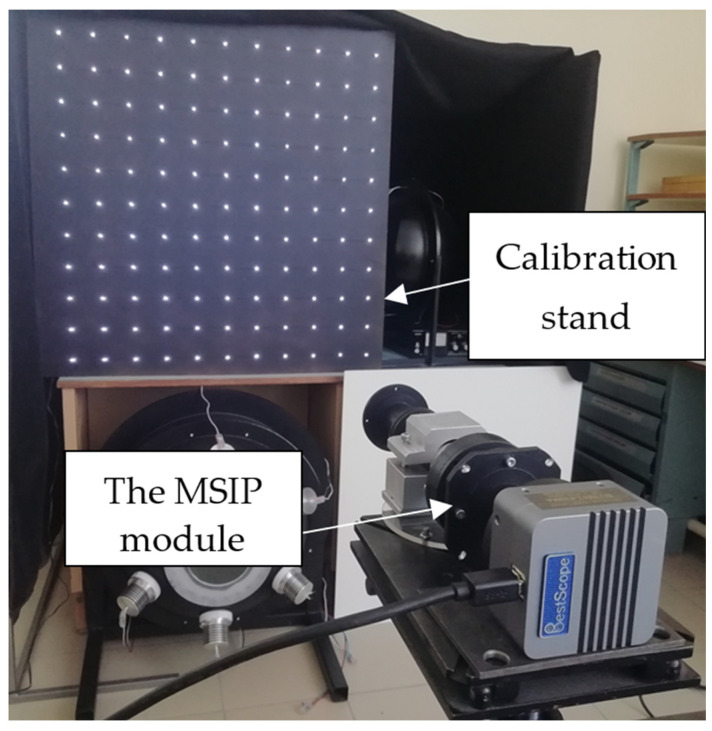
The MSIP module with the stand for geometric calibration.

During the experiments, one of the MSIP polarization modules and a calibration stand with a set of point light sources (LEDs) at the nodes of an imaginary grid were used. A scheme of the experiment is shown in Figure 3.

**Figure 4:**
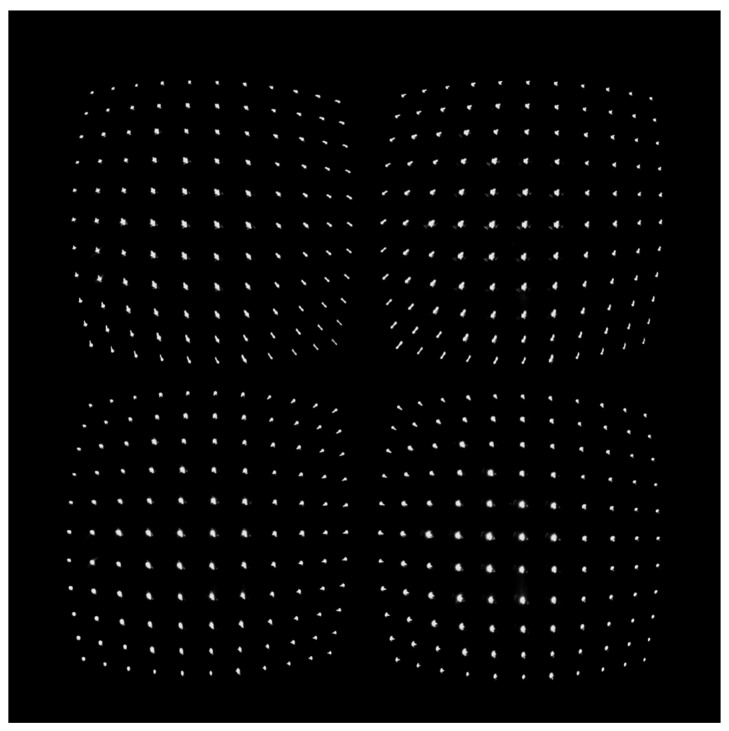
The raw image of the calibration stand captured by the image sensor of the MSIP module. The four sub-images are the images of the calibration stand in the four module channels.

The dimensions of the stand and the distance between the module and the stand were chosen in such a way that the stand completely covered the field of view of the polarimeter module. The images of the stand in the four channels of the module (see Figure 4) were used as input data for the alignment method described above.

The proposed method allowed for the automatic determination of the coordinates of the marker centers on the calibration stand in each of the four images shown in Figure 4 and, thus, the construction of correction models. The image chosen from the first channel of the module (top left sub-image in Figure 4) served as the reference image.

The quality of image alignment in the channels using the obtained correction models was evaluated according to the proposed criteria. The proposed method obtained the alignment and overlay of images from the channels relative to the reference and achieved a standard deviation of marker centers on the calibration stand images, which was accurate to within 0.5 pixels from an initial standard deviation of up to 4.8 pixels. The root mean square error of prediction of the displacement of marker centers was calculated based on the test data and did not exceed 0.5 pixels. In turn, the test data constituted 10% of the total volume of input data used to build the correction models. In general, after alignment of the images, the displacements of the node centers on the images did not exceed 1 pixel.

**Figure 5:**
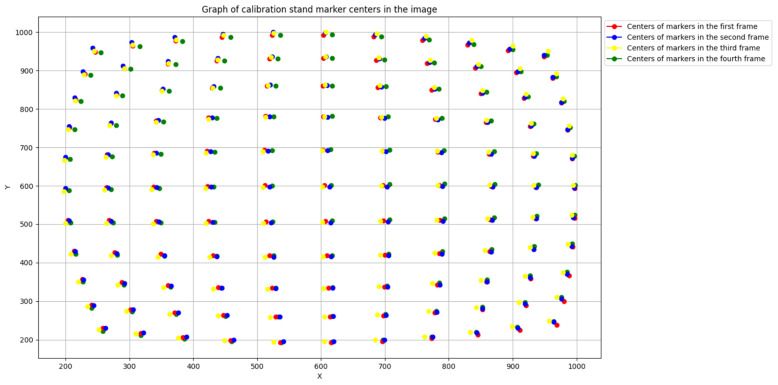
Relative positions of the calibration stand node centers in the images of the polarimeter module channels before alignment.

**Figure 6:**
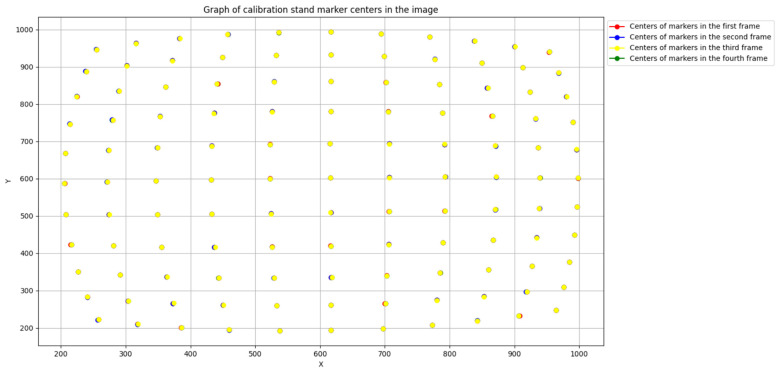
Relative positions of the calibration stand node centers in the images of the polarimeter module channels after alignment.

Figure 5 shows the relative positioning of node centers on the calibration stand images in the channels of the polarimeter module before alignment, while Figure 6 shows the positioning after alignment.

Table 1 presents the coefficient of determination $R^{2}$ for each correction model obtained for image alignment.

As evident from Table 1, each obtained correction model accurately predicted all correction offsets.

Table 2 presents the standard deviation values of the coordinates of marker centers after the image alignment process.

As is evident from Table 2, after the image alignment process, the standard deviation of marker centers for both coordinates did not exceed 0.5 pixels. This indicates a high level of accuracy in image alignment.

Research has shown that increasing the percentage of test data used to evaluate the accuracy of forecasting displacements when using corrective models increases the image alignment error. Therefore, to maintain the high accuracy of corrective models and ensure meaningful accuracy assessment, it was necessary to enhance the calibration setup through introducing independent nodes that do not participate in model construction but are used solely for model accuracy evaluation. This approach not only maintains the high accuracy of the corrective models but also ensures a meaningful accuracy assessment, thereby adding significant value to our research.

Compared with methods for the alignment of images with linear or, especially, radial distortions, which allow for very high-accuracy image alignment (0.05–0.1 pixel), the general method for aligning images with non-linear distortions was not as accurate (0.5 pixel). However, it was twice as accurate as the abovementioned method [43] proposed to compensate for non-linear image distortions. The alignment error of the general method can be reduced by increasing the number of nodes (control points) in the calibration stand. The accuracy of the coordinates of the node centers can also be improved by maximally aligning their brightness in the images obtained in the system channels. This procedure helps to reduce the visible boundaries of the nodes located closer to the center of the stand image and, as a result, reduce the uncertainty in determining the coordinates of their centers. A more accurate determination of the coordinates of the node centers can be expected to lead to more accurate determination of the parameters of the correction model.

## 5. Conclusions

One of the challenges faced when using MCISs is the non-equivalence of the geometric parameters obtained across their channels. This non-equivalence complicates the analysis of the distributions of various informative parameters across observed scenes and reduces the accuracy in estimating their values. The method presented in this article allows for alignment of the geometric parameters of images in MCIS channels through constructing and applying corrective models. These models are built using training data comprising the marker’s center values and their displacements from the reference location in the reference channel obtained from images of a calibration stand. Alignment is achieved through image pre-processing methods, allowing for determination of the marker center’s coordinates on the images.

Through implementing the algorithm in Python, the process of localizing marker bodies on images of the stand, determining the coordinates of marker centers, constructing corrective models, and aligning the geometric parameters of images in MCIS channels was fully automated. This automation significantly reduces the manual effort required, providing relief to researchers.

The application of the developed method to align images from the channels of a real multispectral polarimeter module [67] demonstrated its high accuracy. While the initial root mean square image alignment error was 4.8 pixels for images sized 1200 × 1200, this value did not exceed 0.5 pixels after alignment. The displacements of the marker centers on the calibration stand images did not exceed 1 pixel, indicating the method’s high accuracy. The determination coefficients illustrated that each model effectively predicted all corrective displacements, instilling confidence in its effectiveness.

The proposed method can be used to align the geometric parameters of images in the channels of multi-channel imaging systems with a wide range of geometric distortions, including non-linear ones. It is important to note that the image alignment approach for MCISs used in the proposed method is universal and can be utilized for the channels of any multi-channel imaging system, regardless of its working distance or field of view. The main requirement for using the method is that the calibration image must have clearly defined markers for subsequent analysis, enabling the process of building corrective models to align images in MCIS channels.

## Figures and Tables

**Table 1:** Coefficient of determination $R^{2}$ of the obtained models.

Image Number	$R^{2}$ for X	$R^{2}$ for Y
2	0.983	0.981
3	0.957	0.987
4	0.967	0.962

**Table 2:** The standard deviation of marker centers after the image alignment process.

Image Number	σ for X	σ for Y
2	0.428	0.475
3	0.386	0.434
4	0.472	0.494

## Data Availability

The data presented in this study are available upon reasonable request from the corresponding author.
